# Rowing: extreme physiology and possibility for injury

**DOI:** 10.1186/2052-1847-7-S1-O10

**Published:** 2015-08-11

**Authors:** Henning Bay Nielsen

**Affiliations:** 1 The Copenhagen Muscle Research Centre, Department of Anaesthesia, Rigshospitalet, University of Copenhagen, Denmark

## 

Rowing produces marked changes in oxygen uptake, pulmonary ventilation, cardiac output and lactate with extreme levels for blood acid-base status and pronounced concentration of catecholamines in blood that could affect coagulation. With development of potassiaemia arrhythmia may even be developed that most often may be of supraventricular origin but sudden cardiac death is reported in rowers. Structural myocardial adaptations to intense rowing training that demands the heart to work against high pressure during the stroke need to be considered. Rare cardiac diseases such as the Brugada syndrome and arrhythmogenic right ventricular cardiomyopathy may also provoke cardiac arrest during exercise. The latter is related to genetic disorders but myocarditis could be involved and following rowing the immune system is suppressed. In the subject with vulnerable myocardium abnormal tachycardia may arise during rowing but also bradycardia in the resting period. Furthermore, it is speculated whether silent arrhythmia in combination with dehydration and coagulation disorder could provoke blood clots and even stroke that recently occurred in two Danish athletes. These cases are presented along with parameters indicative of extreme physiology during rowing.

